# Robust inducible Cre recombinase activity in the human malaria parasite *Plasmodium falciparum* enables efficient gene deletion within a single asexual erythrocytic growth cycle

**DOI:** 10.1111/mmi.12206

**Published:** 2013-03-26

**Authors:** Christine R Collins, Sujaan Das, Eleanor H Wong, Nicole Andenmatten, Robert Stallmach, Fiona Hackett, Jean-Paul Herman, Sylke Müller, Markus Meissner, Michael J Blackman

**Affiliations:** 1 Division of Parasitology, MRC National Institute for Medical ResearchMill Hill, London, NW7 1AA, UK; 2 Institute of Infection, Immunity and Inflammation, College of Medical, Veterinary and Life Sciences, University of GlasgowSir Graeme Davies Building, Glasgow, G12 8TA, UK; 3 Wellcome Trust Centre for Parasitology, Institute of Infection, Immunity and Inflammation, College of Medical, Veterinary and Life Sciences, University of GlasgowSir Graeme Davies Building, Glasgow, G12 8TA, UK; 4 CRN2M – UMR 7286, Centre National de la Recherche Scientifique (CNRS), Aix Marseille UniversitéMarseille, France

## Abstract

Asexual blood stages of the malaria parasite, which cause all the pathology associated with malaria, can readily be genetically modified by homologous recombination, enabling the functional study of parasite genes that are not essential in this part of the life cycle. However, no widely applicable method for conditional mutagenesis of essential asexual blood-stage malarial genes is available, hindering their functional analysis. We report the application of the DiCre conditional recombinase system to *Plasmodium falciparum*, the causative agent of the most dangerous form of malaria. We show that DiCre can be used to obtain rapid, highly regulated site-specific recombination in *P. falciparum*, capable of excising *loxP*-flanked sequences from a genomic locus with close to 100% efficiency within the time-span of a single erythrocytic growth cycle. DiCre-mediated deletion of the *SERA5* 3' UTR failed to reduce expression of the gene due to the existence of alternative cryptic polyadenylation sites within the modified locus. However, we successfully used the system to recycle the most widely used drug resistance marker for *P. falciparum*, human dihydrofolate reductase, in the process producing constitutively DiCre-expressing *P. falciparum* clones that have broad utility for the functional analysis of essential asexual blood-stage parasite genes.

## Introduction

The malaria parasite shares its life cycle between a vertebrate host and a mosquito vector, but all the clinical manifestations of malaria are caused by replication of the asexual blood stages within circulating erythrocytes. In the case of *Plasmodium falciparum*, responsible for the most life-threatening form of human malaria, asexual blood-stage forms can be maintained in continuous culture in human erythrocytes (Trager and Jensen, 1976), facilitating study of this important part of the life cycle. Advances in genetic tools for the modification of malarial genes have accelerated understanding of the biology of the parasite, aided within the last decade by the acquisition of the entire genome sequences of *P. falciparum* and several other *Plasmodium* species (e.g. Gardner *et al*., 2002; Pain *et al*., 2008). Because of the relative accessibility of asexual blood stages, these are always used for the introduction of targeting DNA constructs designed for genetic modification by ectopic transgene expression or homologous recombination. Since expression of many *Plasmodium* genes is stage-specific this allows disruption of genes with essential roles restricted to other parts of the life cycle, including the mosquito and exoerythrocytic life cycle stages. In contrast, disruption of parasite genes that are indispensable for blood-stage growth is lethal, preventing the establishment of null mutants and representing a bottleneck in the functional analysis of these genes. In attempts to overcome this, there has been great interest in developing conditional genetic tools suitable for exogenous control of gene expression in *Plasmodium*. One system in which the gene product of interest is fused to a destabilization domain that can be stabilized by small ligands, has been used in *P. falciparum* with some success (Armstrong and Goldberg, 2007; Russo *et al*., 2009; Dvorin *et al*., 2010; Muralidharan *et al*., 2011) but requires that the destabilization tag does not interfere with protein function. Moreover, since the destabilization domain mediates protein degradation by targeting it to the proteasome, the system has limited utility for proteins that are trafficked via the parasite secretory pathway. Tet repressor (TetR)-based regulation of transcription has proven difficult to develop in *Plasmodium* and there are no reports of efficient repression of transcription by TetR in these parasites. The most commonly used tetracycline-sensitive transactivator, a fusion between TetR and the *Herpes simplex* virus VP16 protein, does not activate minimal promoters in the group of apicomplexan parasites to which *Plasmodium* belongs (Meissner *et al*., 2001). An artificial transactivation domain (TATi), when fused to TetR has been successfully used in the related apicomplexan *Toxoplasma gondii* to generate conditional mutants (Meissner *et al*., 2002), and has been used with some success for inducible transgene expression in *P. falciparum* (e.g. Meissner *et al*., 2005; Gilson *et al*., 2008; O'Neill *et al*., 2011). Very recently, functional transactivation domains based on apicomplexan ApiAP2 transcription factors have been elegantly used to obtain stage-dependent tetracycline-dependent gene knock-down in *T. gondii* and *Plasmodium* (Pino *et al*., 1002). Though of great potential, these conditional approaches all require transactivator-responsive minimal promoters that accurately mimic the transcriptional profile of the gene of interest. Despite a promising report describing the use of peptide-morpholino oligomer conjugates in *Plasmodium* (Augagneur *et al*., 2012), most other widely used gene silencing approaches that affect transcript stability, translation or splicing, such as morpholino oligonucleotides, self-cleaving ribozymes and RNA interference (RNAi), have yet to be proven widely effective in *Plasmodium* (Agop-Nersesian *et al*., 2008), in the case of RNAi because of the absence of crucial components of the pathway in the parasite (Baum *et al*., 2009).

Site-specific recombination using phage or yeast-derived recombinases is a method of choice in many models for gene modification or deletion. Two recombinases, Cre and flippase (FLP), have been most widely used. Both recognize short, 34 bp sequences, respectively called *loxP* and *FRT*, and mediate either excision or inversion of intervening sequences depending on the relative orientation of the recognition motifs. FLP has been successfully used in the *Plasmodium berghei* rodent malaria model, exploiting an approach in which developmental stage-specific recombinase activity was obtained by placing FLP under the control of parasite promoters active only in insect stages (Combe *et al*., 2009; Falae *et al*., 2010; Giovannini *et al*., 2011; Lacroix *et al*., 2011). This system has only limited applicability for studying essential asexual blood stage-specific genes. Cre is active in both *P. falciparum* (O'Neill *et al*., 2011) and *T. gondii* (Brecht *et al*., 1999), but attempts to obtain robust regulation of this activity have been unsuccessful, resulting only in constitutive recombinase activity unsuitable for conditional gene modifications. Forms of Cre that can be regulated by hormones or small molecules have been described. In the DiCre system, Cre is expressed in the form of two separate, enzymatically inactive polypeptides, each fused to a different rapamycin-binding protein (either FKBP12 or FRB, the rapamycin-binding domain of the FKBP12-rapamycin-associated protein mTOR) (Chen *et al*., 1995; Choi *et al*., 1996; Liang *et al*., 1999). Rapamycin-induced heterodimerization of the two components restores recombinase activity (Jullien *et al*., 2003;2007). Recent work has shown that this technology functions efficiently in *T. gondii,* with recombination rates of up to 96% upon induction with rapamycin (Andenmatten *et al*., 2013).

Here we show that DiCre provides rapid, highly regulated Cre recombinase activity in *P. falciparum*, capable of excising *loxP*-flanked (floxed) sequences from a genomic locus with close to 100% efficiency within the time-span of a single erythrocytic growth cycle. As proof of principle, we have used the system to recycle one of the most widely used drug selectable markers, in the process producing DiCre-expressing *P. falciparum* clones that will be of great utility for conditional modification of *P. falciparum* genes, including those essential for asexual blood-stage growth.

## Results

### Design of a ‘single vector’ strategy for DiCre-mediated gene knock-down and selectable marker recycling in *P. falciparum*

**Fig. 1:**
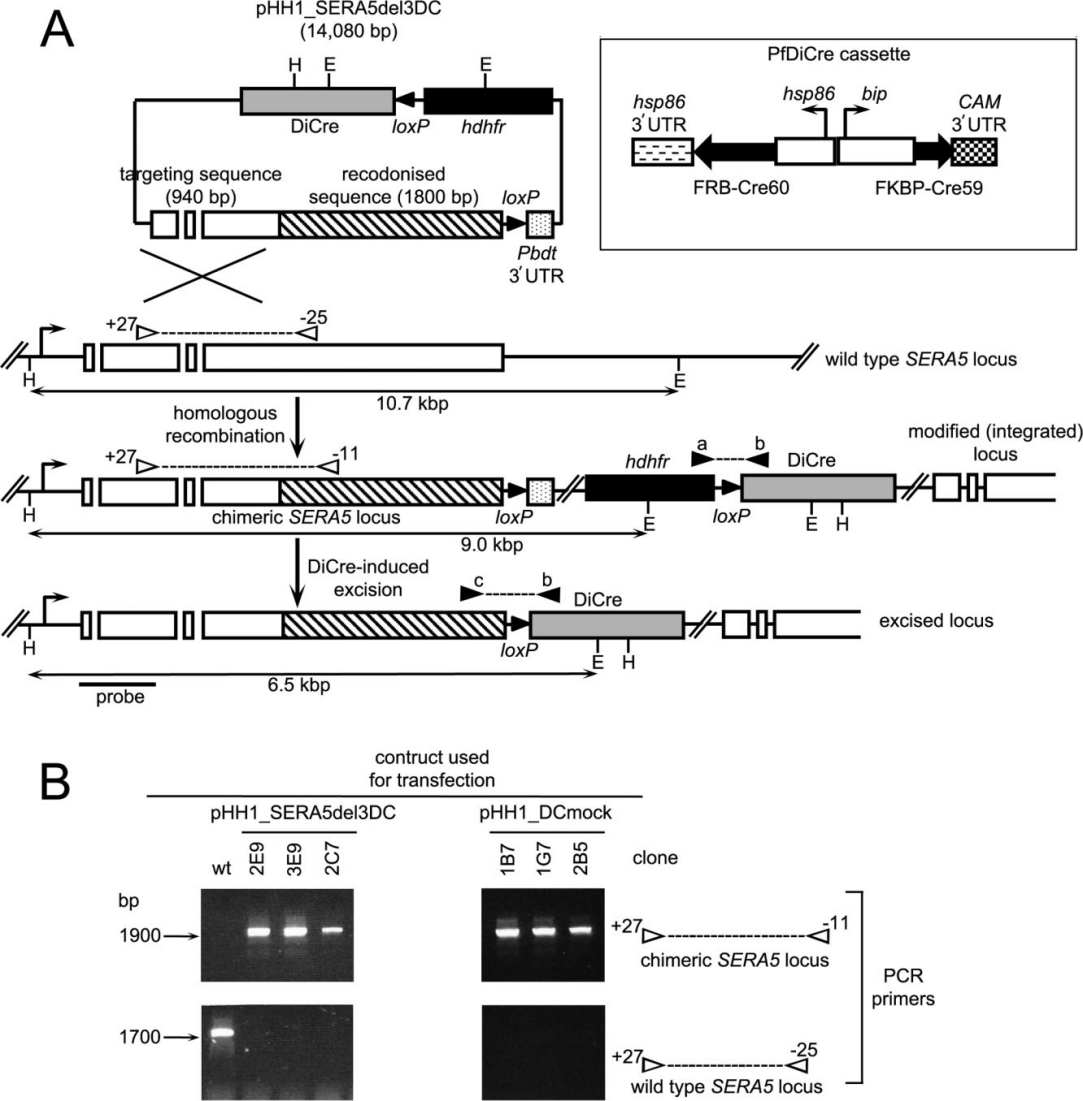
Design of the DiCre targeting vector, predicted homologous integration and recombinase-mediated excision events, and isolation of transgenic *P. falciparum* clones. A. The boxed insert shows an expanded view of the PfDiCre expression cassette. Expression of the DiCre components (black block arrows) is driven by the *P. falciparum* *BiP* and *hsp86* promoters, with transcription termination and polyadenylation being regulated by the 3' UTR sequences from the *P. falciparum* calmodulin (*CAM*) and *hsp86* genes. Main figure: schematic (not to scale) showing the main features of the pHH1_SERA5del3DC plasmid construct. Targeting sequence derived from the native *SERA5* gene was fused in-frame to recodonized sequence (hatched) derived from the *SERA5_synth_* gene. A single EcoNI restriction site (E) within the *hdhfr* cassette (black box), which confers resistance to the antifolate WR99210, is shown. The PfDiCre cassette contains another EcoNI site as well as a unique HindIII site (H). The *P. berghei* *dhfr* 3' UTR (*Pbdt* 3' UTR) lies just downstream of the *SERA5_synth_* sequence in the plasmid. Two *loxP* sites are indicated as black arrowheads in the plasmid. The endogenous *SERA5* locus, which contains three introns, is shown below, as is the expected result of homologous integration of the entire plasmid and the architecture of the modified locus relative to flanking HindIII and EcoNI sites. Episomal plasmids are harboured as concatamers in *P. falciparum*, so integration of more than one copy can occur. However, because the plasmid contains only a partial *SERA5* sequence not preceded by a promoter, the only *SERA5* copy to be transcribed is the modified chimeric gene directly downstream of the endogenous promoter. DiCre-mediated recombination should result in excision of both the *Pbdt* 3' UTR from the modified *SERA5* locus, and the *hdhfr* selectable marker, as well as removal of the entire pHH1_SERA5del3DC plasmid backbone. Note that, even if concatamers of pHH1_SERA5del3DC were to integrate, DiCre-mediated excision would still result in removal of all sequence between the duplicated *loxP* sites, leading to the terminal excised structure shown, in which only a single copy of the modified *SERA5* coding sequence remains under the control of its endogenous promoter, and just a single genomic *loxP* site remains. Positions of primers +27, −11 and −25 used for diagnostic PCR of the expected integration of pHH1_SERA5del3DC and pHH1_DCmock (which is identical aside from the presence of a mock promoter in the place of the *hsp86* and *BiP* promoters within the PfDiCre cassette) are shown by white arrowheads joined by dotted lines. Predicted sizes of the amplicons obtained with primer pairs +27 plus −11, and +27 plus −25, are 1911 bp and 1737 bp respectively. Primer +27 lies just upstream of the targeting sequence in the plasmid, so cannot produce a product from the transfection construct. Positions of primers CAM5‘_R4 (a), hsp86 3'_R1 (b) and sgs5seq5F (c) used for diagnostic PCR analysis of the modified and excised locus are indicated by black arrowheads joined by dotted lines. Predicted sizes of amplicons obtained with primers a plus b, and c plus b are 428 bp and 804 bp respectively. The relative position of the probe used for Southern analysis is indicated. B. Diagnostic PCR analysis of genomic DNA from clones derived by limiting dilution of parasites transfected with constructs pHH1_SERA5del3DC or pHH1_DCmock, confirming integration into the *SERA5* locus as expected. Only wild-type (wt) parasite DNA produced a product with primers +27 plus −25, while the amplicon produced with primer pair +27 plus −11, diagnostic of the expected integration event, was obtained only from the transgenic clones.

X-ray crystal structure analysis of Cre recombinase has shown that it comprises two major helical domains linked by a short, relatively flexible segment (Guo *et al*., 1997). In earlier work, Jullien *et al*. (2007, 2003) showed that the rapamycin-mediated dimerization of two distinct, enzymatically inactive polypeptides approximately corresponding to the individual domains of Cre (residues Thr19–Asn59, called Cre59, and Asn60-343, called Cre60) each fused to a different rapamycin-binding protein (FKBP12 and FRB respectively), resulted in the reconstitution of Cre recombinase activity. Because induction of recombinase activity upon addition of rapamycin does not require *de novo* biosynthesis of the recombinase, induction is very rapid. The N-terminal FKBP12 and FRB fusion partners are linked to their partner Cre sequences through short Gly/Ser-rich linkers, and each protein possesses a nuclear localization signal at its extreme N-terminus. To adapt the DiCre system to *P. falciparum*, we first designed a DiCre cassette in which expression of the FKBP-Cre59 and FRB-Cre60 genes were placed under the control of the strong, constitutive *P. falciparum BiP* and *hsp86* promoters, arranged in a head-to-head orientation (Fig. 1A). For introduction of the expression cassette into the parasite, we decided to incorporate this cassette into a larger targeting vector designed to integrate by homologous recombination into a *P. falciparum* locus in such a way that induction of recombinase activity would produce two distinct effects. First, we wanted to remove the drug selectable marker used to select for the initial integration event. This is because very few drug resistance markers are currently available for experimental genetic modifications in *Plasmodium* (Lin *et al*., 2011), and so the recycling of selectable markers increases flexibility for consecutive gene modifications. Second, we wished to explore the possibility of using a ‘single vector’ strategy to obtain downregulation or knockout of a selected gene of interest (GOI). The 3' UTR of eukaryotic genes generally regulates correct transcription termination and polyadenylation of the mRNA, important for mRNA stability and trafficking. Several previous reports (e.g. Yeoh *et al*., 2007; Combe *et al*., 2009; Giovannini *et al*., 2011) have shown that, whereas the endogenous 3' UTR of *Plasmodium* genes can usually be replaced with other *Plasmodium* 3' UTR sequences without deleterious effects on gene expression, complete removal of the 3' UTR can severely ablate expression levels, effectively resulting in gene knock-down. To investigate the use of DiCre as a means of obtaining conditional gene knock-down in a manner amenable to medium-throughput gene analysis, we sought to use it to obtain conditional removal of the 3' UTR of a GOI. As an initial target GOI we chose *SERA5* (PlasmoDB ID PF3D7_0207600), a member of a family of nine *SERA* genes in *P. falciparum* (Arisue *et al*., 2011). Previous work (Miller *et al*., 2002; McCoubrie *et al*., 2007) has shown that whereas most members of this gene family can be individually disrupted with no phenotypic consequences, the *SERA5* and *SERA6* genes are refractory to disruption using conventional targeted homologous recombination, suggesting that they are indispensable in asexual blood stages of the parasite life cycle. SERA5 is expressed as an abundant, soluble parasitophorous vacuole protein of ∼ 126 kDa, and is thought to play a role in schizont rupture (egress) and/or erythrocyte invasion by released merozoites (reviewed in Blackman, 2008). However its precise function is unknown.

In initial preliminary work we produced construct pHH1SERA5chimWT, designed to integrate by single-crossover homologous recombination into the *SERA5* locus to produce a chimeric gene still under the control of its endogenous promoter and encoding the unmodified SERA5 primary amino acid sequence, but using the 3' UTR from the *P. berghei* dihydrofolate reductase (*dhfr*) gene (*Pbdt* 3' UTR). The use of recodonized sequence within the construct was not essential for this work, but provided the option in future work of introducing desired mutations into this region in the knowledge that single-crossover recombination would preferably occur upstream of the recodonized sequence, as described previously (Child *et al*., 2010; Ruecker *et al*., 2012). Transfection of *P. falciparum* with pHH1SERA5chimWT and selection in the presence of the antifolate drug WR22910 resulted in rapid outgrowth of parasites in which integration of the construct had taken place in the expected manner. Parasite clones obtained by limiting dilution from these lines were phenotypically indistinguishable from wild-type parasites and expressed wild-type levels of SERA5 protein (R. Stallmach and M. Blackman, in preparation), showing that the chimeric *SERA5* gene and appended *Pbdt* 3' UTR were fully functional in maintaining full SERA5 expression levels.

### Rapid and efficient site-specific recombination at a genomic *P. falciparum* locus mediated by induction of DiCre activity

**Fig. 2:**
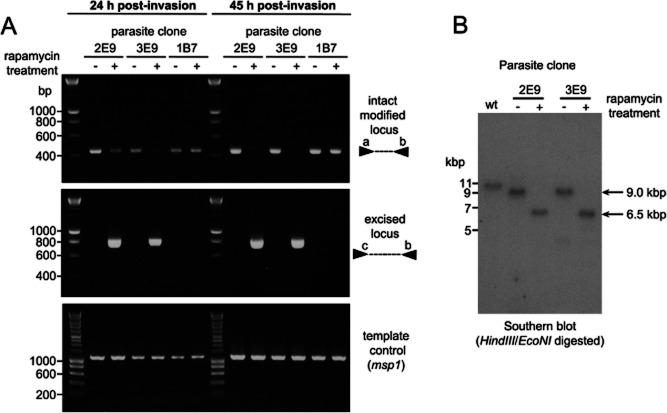
Rapid, regulated and highly efficient DiCre-mediated excision of a floxed genomic *P. falciparum* sequence. A. PCR analysis showing detection of excision following a 4-h-long treatment of clones 2E9, 3E9 or 1B7 with rapamycin at ring stage. Genomic DNA was prepared from the treated parasites at 24 h and 45 h post invasion. Primer pairs a plus b detect the modified locus prior to excision, while primers c plus b detect the appearance of the rearranged locus resulting from site-directed recombination between the *loxP* sites (see Fig. 1A). Primers specific for the *msp1* gene (MSP1_FOR and MSP1_REV; see Table S1) were used as a control to confirm the presence of genomic DNA in all the template samples. B. Southern blots confirming efficient rapamycin-induced excision in the 2E9 and 3E9 transgenic *P. falciparum* clones. The genomic DNA used for the Southern blot was prepared at 45 h post invasion. Phosphorimager quantification of the 6.5 kb ‘excised locus’ signals in the rapamycin-treated 2E9 and 3E9 samples, compared with the position in the same tracks corresponding to the non-excised 9.0 kb species (where no band is visible by eye) showed that excision was 97.9% efficient in the case of clone 2E9, and 97.2% efficient in the case of clone 3E9. However, even upon prolonged exposure, no residual ‘non-excised’ signal was visually detectable in the genomic DNA digests from the rapamycin-treated parasites (not shown).

Encouraged by the above result, we produced constructs pHH1_DCmock and pHH1_SERA5del3DC (Fig. 1A), which used the same targeting sequence as pHH1SERA5chimWT for homologous integration into the *SERA5* locus, but were designed to allow subsequent deletion of the *Pbdt* 3' UTR sequence downstream of the modified *SERA5* gene, along with the human *dhfr* (*hdhfr*) selectable marker, upon induction of DiCre activity. While pHH1_SERA5del3DC contained the complete DiCre cassette shown in Fig. 1A, pHH1_DCmock contained a ‘mock’ cassette in which the *hsp86* and *BiP* promoters were replaced by a single ∼ 930 bp stretch of bacterial coding sequence not expected to drive expression of the DiCre proteins, thereby acting as a negative control for DiCre expression. Transfection of both constructs into *P. falciparum*, followed by limiting dilution cloning of the resulting WR99210-resistant lines, resulted in the isolation of several parasite clones in which integration of pHH1_DCmock or pHH1_SERA5del3DC had taken place (Fig. 1B). To examine the effects of induction of DiCre activity, highly synchronous young ring-stage parasites of clones 2E9 and 3E9 (containing integrated pHH1_SERA5del3DC) and clone 1B7 (containing the integrated ‘mock’ construct pHH1_DCmock) were obtained by adding purified mature schizonts to fresh erythrocytes, allowing invasion to take place for a period of just 2 h, then removing residual schizonts using a combination of Percoll centrifugation and sorbitol-mediated lysis of the schizonts. The resulting cultures, now maintained in the absence of WR99210, were each divided into two and treated for exactly 4 h with either rapamycin (100 nM) or vehicle only (DMSO, 1% v/v). The parasites were then washed, returned to culture in medium lacking WR99210, and sampled at 24 h post invasion (mid-trophozoite stage) and 45 h post invasion (mature schizont stage). Genomic parasite DNA prepared from each time point was then analysed by PCR, using primers diagnostic for either the intact modified locus (primers a and b indicated in Fig. 1A), or the expected genomic product of DiCre-mediated site-specific recombination (primers c and b; Fig. 1A). As shown in Fig. 2A, rapamycin treatment resulted in the efficient conversion of the intact modified *SERA5* locus to the expected excised form of the locus in which deletion of the floxed sequence had taken place (shown in Fig. 1A). Excision was clearly evident by 24 h post invasion, and by 45 h post invasion appeared almost complete, with very little of the intact locus detectable. No excision was detectable by PCR in the 1B7 clone irrespective of whether the parasites had been exposed to rapamycin treatment, indicating a requirement for DiCre expression in the parasite. Of particular importance, no background excision was detectable in 2E9 or 3E9 parasites that had not been exposed to rapamycin, demonstrating robust regulation of DiCre-mediated recombinase activity. To obtain a quantitative measure of excision efficiency, genomic DNA prepared from the 45 h time point was examined by Southern blot (Fig. 2B). This confirmed the high efficiency of the induced site-specific recombination event and allowed it to be calculated by phosphorimager analysis as ∼ 98% by comparison of the intensity of the signals corresponding to the excised and non-excised species in digests of genomic DNA from the rapamycin-treated parasites.

### DiCre-mediated excision of the SERA5 3' UTR fails to silence gene expression due to transcription termination at an alternative cryptic polyadenylation site

**Fig. 3:**
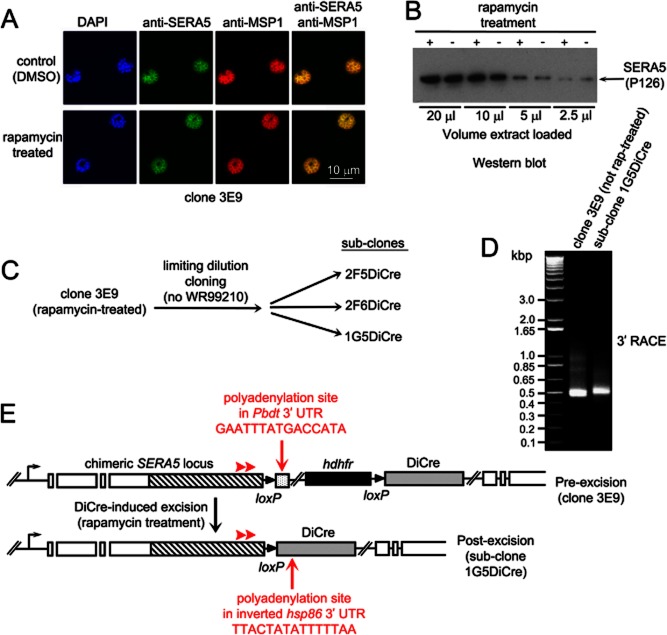
DiCre-mediated excision of the *Pbdt* 3' UTR does not reduce SERA5 expression levels due to the presence of an alternative polyadenylation site within the inverted *hsp86* 3' UTR. A. IFA analysis of clone 3E9 schizonts derived from ring-stage parasites treated for 4 h with vehicle only (DMSO) or 100 nM rapamycin to induce DiCre-mediated excision of the *Pbdt* 3' UTR from the modified *SERA5* locus (see Figs 1 and 2). Parasites were probed with mAbs specific for SERA5, or MSP1 as a control. No change in SERA5 expression levels was detected following rapamycin treatment. 4,6-Diamidino-2-phenylindol (DAPI) was used to detect parasite nuclei. B. Western blot analysis of extracts of the same schizont populations. Different volumes of an SDS extract of intact schizonts were probed with the anti-SERA5 mAb 24C6.1F1. The results confirm no significant difference in SERA5 expression levels between the control and rapamycin-treated parasites. C. Workflow showing isolation by limiting dilution of parasite subclones from rapamycin-treated *P. falciparum* clone 3E9, performed in order to obtain genetically homogeneous parasites harbouring the excised locus architecture. Subclone 1G5DiCre was used for subsequent 3' RACE and transfection analysis. D. Agarose gel electrophoresis of 3' RACE products amplified from total RNA of clone 3E9 and subclone 1G5DiCre. A dominant RACE product was obtained in each case. No product was obtained in the absence of reverse transcriptase (not shown). Sizes in kb of DNA marker fragments (left-hand lane) are indicated. E. Schematic (not to scale) showing the overall structure of the modified *SERA5* locus in clones 3E9 (prior to excision) and subclone 1G5DiCre (following excision), depicted as in Fig. 1. Relative positions of polyadenylation sites identified by sequencing of the cloned 3' RACE products are indicated in red (see Fig. S1 for the aligned sequence data). Positions of *SERA5_synth_* gene-specific forward primers used for the semi-nested 3' RACE are shown (red arrowheads).

The anticipated result of excision of the floxed sequence downstream of the modified *SERA5* locus was to both remove the *Pbdt* 3' UTR, expected to reduce *SERA5* expression levels, and also to excise the *hdhfr* cassette which provides resistance to WR99210 (Fig. 1A). To evaluate the first of these possible phenotypic outcomes, 45-h-old clone 2E9 and 3E9 schizonts from the experiment described above were examined by IFA using a mAb (24C6.1F1) specific for SERA5. Unexpectedly, no discernible difference in fluorescence intensity was evident between parasites that had been exposed to rapamycin at ring stage, and the parallel cultures that had not been rapamycin-treated (Fig. 3A). Given the efficiency of excision demonstrated by PCR and the Southern blot analysis (Fig. 2), this suggested that removal of the *Pbdt* 3' UTR from the modified *SERA5* locus was not sufficient to significantly reduce SERA5 protein expression levels. This was confirmed by Western blot analysis (Fig. 3B).

Although efficient knock-down of *Plasmodium* gene expression has previously been achieved through removal of 3' UTR regulatory sequences (e.g. Giovannini *et al*., 2011), the approach is not uniformly successful. In a recent in-depth investigation of such a case, Ecker *et al*. (1001) showed that FRT-mediated excision of the 3' UTR of the *P. berghei* chloroquine resistance transporter gene (*pbcrt*) failed to ablate gene expression due to the presence of alternative polyadenylation sites at the 3' end of the excised locus, that were efficiently used to stabilize the *pbcrt* mRNA. To address whether a similar phenomenon might explain the continued robust SERA5 expression observed following DiCre-mediated removal of the *Pbdt* 3' UTR in the present study, the rapamycin-treated clone 3E9 parasites were cloned by limiting dilution (in the absence of WR99210) to obtain genetically homogeneous subclones harbouring the excised genomic locus (Fig. 3C). A total of three subclones were expanded and examined by analytical PCR for the presence of the excised genomic architecture, as well as for the presence of the wild-type *SERA5* coding sequence, which could potentially be reconstituted in these transgenic parasites through spontaneous reversion by homologous recombination between the excised modified locus (which lacks the *hdhfr* selectable marker) and the downstream partially duplicated *SERA5* ORF (i.e. the reverse of the single-crossover recombination event depicted in Fig. 1). As expected, all three subclones exhibited the same excised genomic architecture as that in the rapamycin-treated clone 3E9 parasites, with no signs of the intact modified locus and no reversion to the wild-type locus, even after continuous culture for > 2 months (data not shown). One of the subclones (called 1G5DiCre) was then analysed by rapid amplification of cDNA ends (3' RACE), in parallel with the non-rapamycin-treated parental 3E9 clone, using nested forward primers specific for the *SERA5_synth_* gene to examine the 3' structure of the chimeric *SERA5* mRNA transcript(s). As shown in Fig. 3D, there was a clear difference in the size on agarose gel electrophoresis of the dominant 3' RACE products from these two parasite clones, consistent with transcription termination occurring at distinct sites, as expected. To examine this in detail, the 3' RACE products were cloned and sequenced. A comparison of these sequences (Supporting Information Fig. S1) showed that mRNA transcribed from the non-excised chimeric *SERA5* locus underwent polyadenylation as expected at a single major position ∼ 247 bp into the *Pbdt* 3' UTR. In contrast, in the excised locus polyadenylation occurred instead at a position within the inverted *hsp86* 3' UTR which lies immediately downstream of the *SERA5* stop codon in the excised locus (Fig. 3E). No obvious sequence similarity was evident around the polyadenylation sites (Fig. 3E and Fig. S1). Note that the length of the sequenced RACE products was approximately in line with the observed size of the dominant signals observed on gel electrophoresis (∼ 460 bp and ∼ 490 bp for clone 3E9 and subclone 1G5DiCre respectively), suggesting that the sequenced clones were representative of the major RACE products in each case. These observations strongly suggest that the *hsp86* 3' UTR sequence (which was present in the modified locus in order to regulate transcription of the FRB-Cre60 component of the DiCre cassette; see Fig. 1 insert), possesses bidirectional transcription termination and polyadenylation functionality. Collectively, our results explain the observed lack of SERA5 knock-down upon removal of the *Pbdt* 3' UTR.

### Efficient recycling of the hdhfr selectable marker through DiCre-mediated excision, and production of DiCre-expressing ‘recipient’ *P. falciparum* clones

**Fig. 4:**
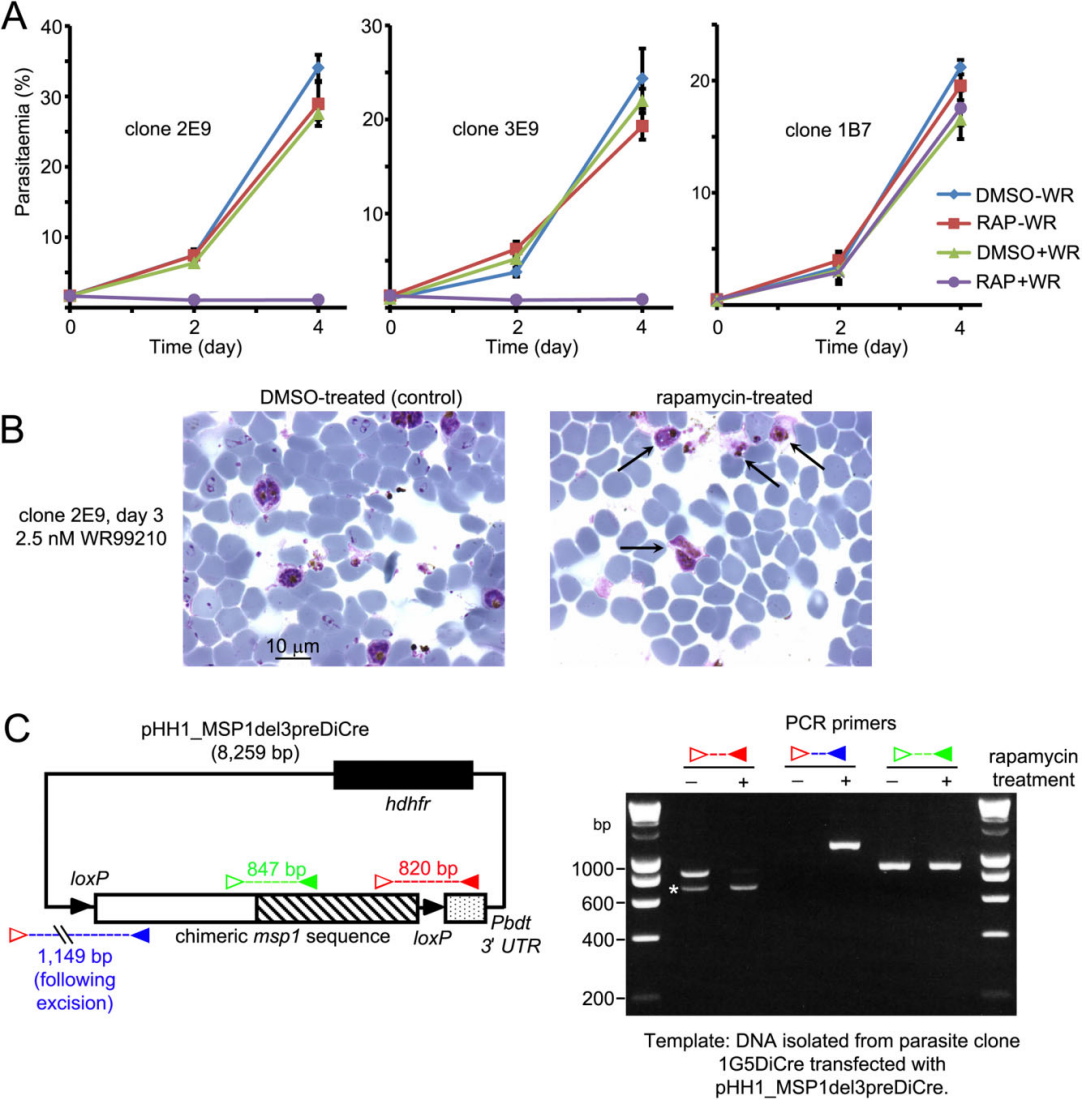
DiCre-mediated excision successfully recycles the *hdhfr* selectable marker and produces a *P. falciparum* recipient clone that constitutively expresses DiCre. A. Typical growth assay showing the results of expanding the control (DMSO-treated) or rapamycin-treated clones 2E9, 3E9 and 1B7 in the presence or absence of WR99210. As predicted based on the efficient excision of the *hdhfr* cassette, the rapamycin-treated 2E9 and 3E9 clones did not replicate in medium containing WR99210. In contrast, rapamycin treatment of clone 1B7, which harbours the ‘mock’ DiCre cassette, had no effect on growth under any conditions. Day 0 corresponds to the point (∼ 60 h following treatment with rapamycin or DMSO) at which the cultures were transferred to fresh medium ± WR99210. Each data point represents the mean of triplicate microscopic counts, each of at least 500 cells. Error bars, ± 1 SD. B. Giemsa-stained microscopic images of clone 2E9 at day 3 following the start of culture in the presence of WR99210. The appearance of the poorly staining, dysmorphic schizonts (arrowed) in the rapamycin-treated cultures is typical of that usually observed after WR99210 treatment of drug-susceptible wild-type parasites. By day 4, these had completely degenerated and disappeared from the cultures. In contrast, the DMSO-treated control cultures contain numerous healthy schizonts and young ring stages, indicative of normal levels of growth. C. Induction of DiCre-mediated excision in the 1G5DiCre recipient subclone. Shown (left-hand side) is a schematic of reporter plasmid construct pHH1_MSP1del3preDiCre (not to scale) alongside results of PCR analysis of DNA isolated from 1G5DiCre parasites harbouring the plasmid, following treatment ± rapamycin. Rapamycin treatment induced efficient excision of the floxed *Pbdt* 3' UTR and *hdhfr* cassette from the plasmid, proving expression of DiCre from the genomic cassette incorporated into the modified *SERA5* locus of the 1G5DiCre parasites. No excision was observed in the absence of rapamycin. The relative positions of primers used for the PCR are indicated as coloured arrowheads. Primers used were: 3D7synMSP1_FOR2 (open red); PbDT3'5’R1 (solid red); 3D7endoMSP1_REV4 (blue); 3D7endoMSP1FOR1 (open green); and 3D7synMSP1_REV3 (solid green). See Table S1 for primer sequences. The hatched region in the pHH1_MSP1del3preDiCre schematic indicates the synthetic (recodonized) *msp1* sequence, and the expected sizes of the various amplicons are indicated. The extreme left-hand and right-hand tracks on the gel contain DNA size markers. The ∼ 700 bp fragment (asterisked) present in the 3D7synMSP1_FOR2 (open red) plus PbDT3'5’R1 (solid red) PCR tracks is a product of mis-priming, since this was also amplified from 1G5DiCre parasites that had not been transfected with plasmid pHH1_MSP1del3preDiCre (not shown). Rapamycin treatment results in loss of the 820 bp PCR product and the appearance of an excision-specific 1149 bp product. Note that under the PCR conditions used, no product was expected to be amplified from the intact pHH1_MSP1del3preDiCre with primers 3D7synMSP1_FOR2 (open red) and 3D7endoMSP1_REV4 (blue) due to the large size of that predicted product.

To address the second predicted outcome of DiCre-mediated excision from the modified *SERA5* locus – removal of the *hdhfr* selectable marker cassette – schizonts from all three clones 2E9, 3E9 and 1B7 treated with or without rapamycin as described in Fig. 2 were allowed to undergo invasion overnight in the absence of WR99210, to form a new generation of ring-stage parasites. Each culture was then divided into two, and culture continued in either the presence or absence of WR99210. As shown in Fig. 4A and B, whereas rapid expansion of the non-rapamycin-treated 2E9 and 3E9 clones took place in both the presence and absence of WR99210, those 2E9 and 3E9 parasites that had been rapamycin-treated at ring stage in the first cycle instead displayed extensive cell death in the presence of WR99210, consistent with the removal of the *hdhfr* cassette in the great majority of the population. In contrast, and again consistent with the excision results, the 1B7 clone parasites continued to grow well, irrespective of whether they had been rapamycin treated, or of the presence of WR99210. These data convincingly support the PCR and Southern blot results, showing efficient removal of the *hdhfr* resistance marker in the 2E9 and 3E9 parasites upon rapamycin-induced, DiCre-mediated excision of the floxed genomic sequence.

Prolonged culture (> 28 days) of the rapamycin-treated 2E9 and 3E9 parasites in the presence of WR99210 resulted in the eventual outgrowth of drug-resistant parasites (not shown). We reasoned that these surviving parasites (referred to as 2E9WR and 3E9WR) likely represented a very small minority of the original parasite clones that had either not undergone DiCre-mediated excision upon the first round of rapamycin treatment, or had undergone anomalous excision. To test this prediction, the expanded 2E9WR and 3E9WR parasite lines were synchronized then subjected to a second 4 h treatment ± rapamycin, and their genomic structure analysed again by PCR 40 h later. For comparison these experiments were performed in parallel with similar treatments of the original 2E9 and 3E9 clones that had not previously been exposed to rapamycin. As shown in Fig. S2, whereas the original 2E9 and 3E9 clones exhibited efficient rapamycin-dependent excision as seen previously, the WR99210-resistant parasite lines showed either poor excision (3E9WR) or good excision but with signs of pre-existing excision (2E9WR) which presumably had failed to remove the *hdhfr* cassette, leaving these parasites WR99210-resistant. While these results cannot be fully explained without detailed genomic sequencing of the modified loci, they were consistent with the recovered WR99210-resistant parasites being the results of very low-level anomalous DNA rearrangements that were not typical of the great majority of the rapamycin-treated 2E9 and 3E9 parasites.

To finally demonstrate the utility of our approach for producing a DiCre-expressing recipient parasite line with potential for further gene manipulation, we returned to a more detailed analysis of subclone 1G5DiCre, which was derived as described above by limiting dilution cloning (in the absence of WR99210) from clone 3E9 parasites that had been rapamycin-treated to induce excision of the *hdhfr* cassette. To test for expression of DiCre from the integrated genomic DiCre cassette in these parasites, subclone 1G5DiCre was transfected by electroporation with a reporter construct called pHH1_MSP1del3preDiCre (Fig. 4C). This construct contains a *hdhfr* selectable marker cassette and a chimeric *msp1* gene fragment, followed by the *Pbdt* 3' UTR and flanked by *loxP* sites; importantly, it lacks a DiCre cassette, so excision of the floxed sequence can only take place if the 1G5DiCre parasites continue to express DiCre from the integrated genomic DiCre cassette. 1G5DiCre parasites not subjected to electroporation, or mock-transfected (i.e. subjected to electroporation in the absence of the transfection construct), rapidly died upon culture in the presence of WR99210, as expected (data not shown). In contrast, drug-resistant parasites appeared in the pHH1_MSP1del3preDiCre-transfected 1G5DiCre cultures within 6 days. These parasites were expanded, synchronized, then treated ± rapamycin for 4 h as described above and analysed 40 h later by PCR, using primers designed to detect excision of the floxed sequence, as well as control primers. As shown in Fig. 4C, rapamycin treatment resulted in efficient excision of the floxed sequence from the episomal pHH1_MSP1del3preDiCre construct, proving robust constitutive expression of DiCre in the 1G5DiCre parasites. These results confirm the usefulness of the 1G5DiCre parasite subclone as a recipient line for future DiCre-mediated mutational analysis of *P. falciparum* genes.

## Discussion

Despite the medical importance of *P. falciparum*, and in particular of its asexual blood-stage life cycle, obtaining conditional regulation of gene expression in this organism has proven extremely challenging. The short erythrocytic life cycle of *P. falciparum* (∼ 48 h) raises particular problems in this regard; in order to observe developmental stage-specific phenotypic changes at the population level in a conditional mutant over the course of a single erythrocytic cycle – especially important if the outcome of mutagenesis is lethal – switch-off of gene expression needs to be both efficient and rapid. We have shown here that the DiCre system meets both those critical requirements, allowing rapid, highly regulated and remarkably efficient site-specific recombination within the course of a single erythrocytic growth cycle. We designed our strategy to simultaneously impose two measurable effects on gene expression in our transgenic clones. First we attempted knock-down of *SERA5* expression by removal of its introduced *Pbdt* 3' UTR, anticipating that this would result in lowered protein expression levels by reducing mRNA stability. We were surprised to observe no detectable change in SERA5 expression levels. SERA5 is expressed at extraordinarily high levels in asexual blood-stage schizonts, and indeed is probably one of the most abundant schizont-stage parasite proteins (Lasonder *et al*., 2002; Le Roch *et al*., 2004). Small alterations in *SERA5* mRNA stability may therefore have little effect on overall protein levels. We reasoned that an alternative explanation for the lack of alteration in SERA5 levels might be the presence of a cryptic 3' UTR in the apposed *hsp86* 3' UTR, which was shifted adjacent to the *SERA5* stop codon following DiCre-mediated excision from the modified locus (Fig. 1A), even though this was in the opposite orientation to that previously known to mediate correct transcription termination. Examination of subclone 1G5DiCre, derived from the rapamycin-treated 3E9 parasites, confirmed this suspicion; 3' RACE analysis identified a polyadenylation site in the inverted *hsp86* 3' UTR at a position that shows no obvious sequence similarity to that identified in the authentic *Pbdt* 3' UTR, which is widely used to regulate transcription of transgenes in both *P. falciparum* and *P. berghei*. This suggests that the *hsp86* 3' UTR contains bidirectional transcription termination and polyadenylation signals. Interestingly, a recent report from Ecker *et al*. (1001) made a very similar observation; following unsuccessful attempts to obtain knock-down of the *pbcrt* gene in *P. berghei* those authors identified efficient polyadenylation sites in the inverted 3' UTR from the *P. berghei* thrombospondin related adhesive protein (*trap*) gene, which was translocated to a position immediately downstream of the *pbcrt* gene upon FLP-mediated excision. Together, these findings show that great caution should be exercised in the design of constructs intended for removal of 3' regulatory sequences as a route to gene knock-down in *Plasmodium*. Certainly, in the light of our results future work will focus on alternative approaches to DiCre-mediated *SERA5* knockout, including truncation of the coding sequence by inserting the upstream *loxP* site into one of the 5'-proximal introns within the *SERA5* gene, or indeed floxing the entire locus. This would likely require the use of a double-crossover strategy to incorporate the two *loxP* sites, a feasible approach in *P. falciparum* (Duraisingh *et al*., 2002).

The second intended outcome of DiCre-mediated excision of the modified SERA5 locus, removal of the *hdhfr* selectable marker cassette, resulted as expected in a dramatic reversal of resistance of the 2E9 and 3E9 clones to WR99210, consistent with the high efficiency of site-specific recombination determined by PCR and Southern blot analysis. Importantly, background excision was undetectable by PCR in the absence of rapamycin treatment, demonstrating stringent regulation of DiCre activity. The result demonstrates robust, conditional deletion of what is effectively an essential parasite transgene in the presence of the antifolate drug. It also demonstrates the ease with which the DiCre system can be used to recycle selectable markers, only a limited number of which are available for use in *Plasmodium*. This is crucial for enabling consecutive genetic manipulations, required for disruption of multiple genes at different loci, or complementation of knockouts. Three parasite subclones derived from the rapamycin-treated 3E9 clone were found to be genetically stable, and detailed examination of subclone 1G5DiCre showed it to be readily transformed back to WR99210-resistance by transfection with a reporter plasmid containing the *hdhfr* cassette, confirming recycling of the selectable marker. In addition we confirmed constitutive expression of DiCre in the parasites by demonstrating rapid excision of a floxed sequence from the input plasmid upon rapamycin treatment of the transfected parasites. The DiCre-expressing ‘recipient’ *P. falciparum* clones will be invaluable tools for further gene targeting studies, enabling the use of much smaller targeting constructs than that described here, since the constructs will no longer need to incorporate the ∼ 5.5 kb DiCre cassette (which is now integrated into the genome of the recipient parasite clones).

The DiCre strategy presented here will have important applications in other *Plasmodium* species, including the human pathogen *Plasmodium knowlesi*, which has recently been adapted to continuous culture in human erythrocytes (Moon *et al*., 2013). It also has great potential for use in rodent malaria models such as the widely used and highly genetically tractable species *P. berghei*. DiCre has previously been shown to be inducible *in vivo* using rapamycin (Jullien *et al*., 2007), and rapamycin is used clinically as an immunosuppressive agent, even accumulating strongly in erythrocytes following administration (Yatscoff *et al*., 1995; Trepanier *et al*., 1998). We would therefore expect that it should be possible to induce DiCre activity in circulating blood-stage rodent parasites by parenteral administration of rapamycin. As an alternative approach, and to avoid potential experimental complications introduced by the immunosuppressive and antiproliferative activities of rapamycin in mice, isolated parasites (which can be maintained transiently *ex vivo*) could be treated briefly *in vitro* with a ‘pulse’ of rapamycin to induce DiCre activity, then simply washed and reintroduced into the animal. Rapamycin does have antimalarial properties, possibly through binding the parasite FKBP homologue (Monaghan and Bell, 2005); however, the reported IC_50_ value of rapamycin against *P. falciparum* (2.6 μM; Bell *et al*., 1994) is much higher than the concentration used here to induce DiCre activation (100 nM), and in accord with that we observed no deleterious effects on parasite growth following brief treatment with the drug. The availability of a series of rapamycin analogues (‘rapalogues’) some of which bind certain FRB or FKBP12 mutants well, but have lowered affinity for endogenous mammalian FKBP12 or FRAP (Clackson *et al*., 1998; Pollock and Clackson, 2002; Bayle *et al*., 2006), provides even more potential flexibility for use of DiCre *in vitro*. Unfortunately several of these compounds are not suitable for use in mice as they appear to be rapidly cleared *in vivo* (Jullien *et al*., 2007). This limitation may be overcome by a second-generation ‘DiCre2’ system currently under development in which both DiCre components are fused to an FKBP mutant which can be dimerized by the rapalogue AP20187; preliminary studies have indicated that this is effective *in vivo* with no physiological side-effects (Herman and Jullien, 2012; also J.-P. Herman and C. Monetti, unpublished). We expect the DiCre strategy to have a marked impact on understanding of gene function in the malaria parasite and other apicomplexan pathogens.

## Experimental procedures

### Reagents and antibodies

Rapamycin was obtained from Sigma, UK (catalogue number R0395). Stock solutions (10 μM) were prepared in DMSO and stored in aliquots at −20°C. The antifolate drug WR99210 was from Jacobus Pharmaceuticals (New Jersey, USA). Monoclonal antibody (mAb) X509, which recognizes the parasite major merozoite surface protein MSP1, has been described previously (Blackman *et al*., 1991), as has the anti-SERA5 mAb 24C6.1F1 (Delplace *et al*., 1985), which was a kind gift of Jean-Francois Dubremetz (University of Montpellier 2, France).

### Parasite cultures and transfections

Asexual blood-stage cultures of *P. falciparum* clone 3D7 were maintained *in vitro* and synchronized according to standard procedures (Blackman, 1994; Yeoh *et al*., 2007) in RPMI 1640 medium containing the serum substitute Albumax (Invitrogen). For introduction of transfection constructs, mature schizonts were enriched from highly synchronous cultures using Percoll (GE Healthcare) as described previously (Harris *et al*., 2005), and transfected by electroporation with 10 μg of circular plasmid DNA using the Amaxa 4D electroporator (Lonza) and the P3 Primary cell 4D Nucleofector X Kit L (Lonza) and program FP158, exactly as recently described for *P. knowlesi* (Moon *et al*., 2013). Selection for parasites harbouring the plasmid was performed by culture in medium containing 2.5 nM WR99210. Selection for parasites in which integration of transfected DNA into the genome had taken place was promoted by cycles of culture in the absence and presence of WR99210, as described previously (Harris *et al*., 2005). When integration was detected by diagnostic PCR, integrant clones were obtained by limiting dilution and maintained in medium containing WR99210. Parasite growth rates were assessed by microscopic examination of Giemsa-stained thin films at 2-day intervals, and expressed as percentage parasitaemia (percentage of parasitized erythrocytes in the population)

### Production of *P. falciparum* transfection vector pHH1SERA5chimWT

A synthetic, recodonized *SERA5* gene (*SERA5_synth_*), codon-optimized for expression in *Escherichia coli* and based on the predicted *P. falciparum* 3D7 sequence (PlasmoDB ID PF3D7_0207600) was synthesized by GeneArt AG (Regensburg, Germany) and provided with terminal 5’ SalI and 3’ XhoI sites. A 940 bp targeting sequence including the second and third intron of the *SERA5* gene (PlasmoDB ID PF3D7_0207600) was amplified by PCR from *P. falciparum* 3D7 genomic DNA using the oligonucleotide pair +S5endogHpaI and −S5endogClaI (see Supporting information Table S1 for all primer sequences used in this work). A 3'-segment of the recodonized *SERA5_synth_* gene was amplified by PCR using the oligonucleotide pair +S5Seq1021 and −S5stopXhoI. The PCR amplicons, of 957 bp and 2007 bp respectively, were ligated into pCR-Blunt using the Zero Blunt PCR Cloning Kit (Invitrogen). Clones with a suitable insert orientation were selected and then the endogenous *SERA5* targeting sequence was ligated in frame to the 5' end of the 3' segment of the *SERA5_synth_* gene, using restriction sites KpnI (derived from the pCR-Blunt multiple cloning site) and ClaI. The entire chimeric sequence was then excised with HpaI and XhoI and ligated into pHA3.HH1 (Yeoh *et al*., 2007) to produce pHH1SERA5chimWT. This final construct comprised 940 bp of authentic *SERA5* sequence (the targeting sequence), fused in frame to 1800 bp of recodonized synthetic *SERA5_synth_* sequence encoding the remaining 3' region of the *SERA5* ORF, followed by a stop codon, an XhoI site, then a 3HA epitope tag sequence, and another stop codon, all directly upstream of the 3' UTR from the *P. berghei* dihydrofolate reductase (*dhfr*) gene (*Pbdt* 3' UTR).

### Production of the PfDiCre cassette

PCR amplification was conducted using AccuPrime™ Pfx SuperMix (Invitrogen, UK) and when required PCR products were subcloned into the pScB subcloning vector (Aligent Technologies, UK). A ‘mock’ promoter region was amplified from *E. coli* genomic DNA using primers EWmock1 and EWmock2. The resulting amplicon (corresponding to ∼ 930 bp of the *E. coli pmba* gene, GenBank/EMBL Accession No. X54152) was subcloned into pScB. Restriction digestion of the intermediate vector using PstI and HindIII released the mock promoter fragment which was subsequently cloned into the pBlueScript SK+ phagemid (GenBank/EMBL Accession No. X52328) pre-digested with PstI and HindIII, to form pmockINT. The FKBP-Cre59 gene was amplified using primers EWCRE59For and EWCRE59Rev from plasmid TUB8FKBP-Cre59-HX (Andenmatten *et al*., 2013), and subcloned into PstI and EcoRI-digested pmockINT to create pCre59INT. The 3' UTR of the *P. falciparum* calmodulin gene (PfCAM) was amplified from *P. falciparum* genomic DNA using primers EWCAM3For and EWCAM3Rev. The PCR fragment was subcloned into pScB. To excise the 3' CAM fragment, the intermediate vector was digested with NotI and PstI and inserted into pCre59INT to form the pCre59 vector. The FRB-Cre60 coding sequence was amplified by PCR using primers EWCRE60For and EWCRE60Rev from plasmid TUB8FRBCre60-HX (Andenmatten *et al*., 2013). The amplicon was subcloned into pScB, released using HindIII and KpnI and ligated into the pCre59 vector to form pCre59/Cre60INT. Finally, the *hsp86* 3' UTR was amplified by PCR from *P. falciparum* genomic DNA using primers EWHSP863For and EWHSP863Rev, cloned into pScB, released with ClaI and KpnI and ligated into pCre59/Cre60INT to create the ‘mock’ pCre59/Cre60 DiCre vector called (DiCre24A). To obtain expression of DiCre in *P. falciparum*, the mock promoter in this vector was replaced with the *P. falciparum hsp86* and *BiP* promoters, arranged in a head-to-head orientation. To do this, the *hsp86* 5' flanking region was amplified from pA289-attP-BSD (a kind gift of Andy Osborne, University College London, UK) using primers hsp86 5'_F1 and hsp86 5'_R1. The resulting product was cloned into DiCre24A using EcoRI and HindIII restriction sites, replacing the mock promoter region and giving rise to plasmid pBS_DC_hsp86. The *BiP* 5' UTR was amplified from pHH4 using primers bip_F1 and bip_R1, and cloned into pBS_DC_hsp86 using AfeI and EcoRI, giving rise to the PfDiCre expression cassette vector pBS_DC_hsp86/Bip5'.

### Production of *P. falciparum* transfection vector pHH1_SERA5del3DC

This construct was designed to integrate by single crossover homologous recombination into the *SERA5* locus in such a manner that DiCre-mediated excision from the modified locus would remove its introduced 3' UTR (*Pbdt* 3' UTR), as well as removing the *hdhfr* selectable marker cassette from the modified locus. Cloning of other target gene sequences into the transfection plasmid can be carried out using a restriction site in the multiple cloning site (MCS) and the unique XhoI site immediately downstream of the recodonized *SERA5* sequence (with or without a stop codon depending on whether the 3HA epitope tag is required) or the unique AvrII site.

PCR reactions were performed using pHH1SERA5chimWT as template, and primers sgS5seq4F and 3HA_AvrII_LoxP or AvrII_LoxP_PbDT3'_F and NotI_PbDT3'_R. Products from these reactions were mixed in equal amounts and an overlapping PCR carried out using primers sgS5seq4F and NotI_PbDT3'_R. The resulting amplicon was cloned into pHH1SERA5chimWT using the XhoI and NotI restriction sites, generating plasmid pHH1_sera5_LoxP1. This now contained an AvrII and a *loxP* site between the 3HA tag and *Pbdt* 3' UTR sequences. To incorporate the *loxP* site and a MCS (comprising SpeI, SnaBI, AflII and HpaI sites) upstream of the *SERA5* targeting gene sequence in this plasmid, three sequential rounds of PCR amplification were carried out using forward primers (i) MCS_HpaI, (ii) U1_MCS and (iii) LoxP_MCS_XL and reverse primer EndoS5_R1. The resulting amplicon was blunt ended with T4 DNA polymerase then digested with BstZ171 and cloned into pHH1_sera5_LoxP1 pre-digested with HpaI and BstZ171, giving rise to plasmid pHH1_PreDiCre_A. This was digested with EcoRI, blunted with T4 DNA polymerase and re-ligated to remove the EcoRI site, giving rise to 3A_ΔEcoRI. This was digested with HindIII, blunted with T4 DNA polymerase and re-ligated to remove the HindIII site, giving rise to pHH1_PreDC_A_ΔH/ΔE. The entire DiCre cassette from plasmids DiCre24A or pBS_DC_hsp86/Bip5' was finally cloned into pHH1_PreDiCre_A and pHH1_PreDC_A_ΔH/ΔE respectively, using the SpeI and AflII restriction sites, to produce the final transfection constructs, pHH1_DCmock and pHH1_SERA5del3DC.

### Production of *P. falciparum* reporter transfection vector pHH1_MSP1del3preDiCre

This construct was used to assay for inducible DiCre activity in the 1G5DiCre *P. falciparum* subclone. Briefly, it is identical to pHH1_PreDiCre_A described above except that the chimeric *SERA5* sequence was replaced with ∼ 990 bp of *msp1* sequence fused in frame to ∼ 1400 bp of recodonized *msp1* sequence. The plasmid therefore contains two *loxP* sites flanking the *Pbdt* 3' UTR and *hdhfr* cassette. Full details of production of this construct will be provided in a later paper (S. Das and M. Blackman, in preparation).

### Indirect immunofluorescence (IFA) and Western blot

IFA and Western blot analysis using mAbs 24C6.1F1 and X509 were performed as described previously, using SDS-extracts of mature intact Percoll-enriched schizonts for the Western blot analysis (Jean *et al*., 2003), and formaldehyde-fixed thin films of cultures containing mature schizonts for the IFA (Harris *et al*., 2005; Ruecker *et al*., 2012).

### Southern blot

For Southern blot analysis, a 597 bp probe corresponding to endogenous *SERA5* coding sequence lying just upstream of the targeting sequence in constructs pHH1SERA5chimWT, pHH1_A_DC_mock and pHH1_3A_H + B_ΔH/E_SERA5 (which was identical in all cases) was produced by PCR amplification from *P. falciparum* 3D7 genomic DNA with primers SERA5_US_F and SERA5_US_R (Table S1). Radiolabelling of the probe and hybridization to HindIII/EcoNI-digested genomic DNA from parasite clones of interest was performed as described previously (Ruecker *et al*., 2012). Quantification of the conversion of the signal corresponding to the non-excised modified *SERA5* locus to the excised form was performed by phosphorimager analysis on a STORM 860 Molecular Imager (GE Healthcare) using ImageQuant software.

### 3' Rapid amplification of cDNA ends (3' RACE)

Total RNA from the 3E9 *P. falciparum* clone (non-rapamycin-treated) and the 1G5DiCre subclone was prepared using a TRIzol Plus RNA isolation kit. First-strand cDNA synthesis was conducted using 3 μg of total RNA, oligo dT adapter primers and SuperScript II reverse transcriptase, using a 3' RACE kit (Invitrogen) according to the manufacturer's protocol. Subsequently, semi-nested PCR reactions were performed to specifically amplify cDNA derived from polyadenylated *SERA5* mRNA, using *SERA5_synth_*-specific primers SERA5_3R1 and SERA5_3R2 (Table S1) as first and second gene-specific primers. For both PCR reactions AUAP from the 3' RACE kit was used as the reverse primer. PCR products were purified (Qiagen MinElute kit) and cloned into the pGEM T-Easy vector (Promega) for nucleotide sequencing.
