# Supplementary material for: Robust inducible Cre recombinase activity in the human malaria parasite Plasmodium falciparum enables efficient gene deletion within a single asexual erythrocytic growth cycle
Source: Mol Microbiol. 2013 Mar 26;88(4):687–701. doi: 10.1111/mmi.12206 (PMC3708112; doi:10.1111/mmi.12206)
Supplement: Supplementary file 1 [file mmi0088-0687-SD1.pdf]

Supporting information

Robust inducible Cre recombinase activity in the human malaria parasite *Plasmodium falciparum* enables efficient gene deletion within a single asexual erythrocytic growth cycle

Christine R Collins[1]**, Sujaan Das[1]**, Eleanor H Wong[2], Nicole Andenmatten[3], Robert Stallmach[1], Fiona Hackett[1], Jean-Paul Herman[4], Sylke Müller[2], Markus Meissner[3]*, and Michael J Blackman[1]*

[1]Division of Parasitology, MRC National Institute for Medical Research, Mill Hill, London NW7 1AA, UK.

[2]Institute of Infection, Immunity and Inflammation, College of Medical, Veterinary and Life Sciences, Sir Graeme Davies Building, University of Glasgow, Glasgow G12 8TA, UK.

[3]Wellcome Trust Centre for Parasitology, Institute of Infection, Immunity and Inflammation, College of Medical, Veterinary and Life Sciences, Sir Graeme Davies Building, University of Glasgow, Glasgow G12 8TA, UK.

[4]CRN2M - UMR 7286, Centre National de la Recherche Scientifique (CNRS), Aix Marseille Université, Marseille, France.

*For correspondence. Email Markus.Meissner@glasgow.ac.uk or mblackm@nimr.mrc.ac.uk; Tel: +44 208 816 2127; Fax: +44 208 816 2730.

**These authors contributed equally to this work.

Fig. S1. Identification by 3' RACE of a cryptic polyadenylation site in the inverted *hsp86* 3' UTR. Shown are the entire nucleotide sequences of cloned 3' RACE products amplified from total RNA of the 3E9 *P. falciparum* clone (top) and the 1G5DiCre sub-clone (bottom), aligned in each case with the corresponding DNA sequence. Sequences of 2 distinct clones are shown in each case. The RACE products comprise the extreme 3' 46 bp of the *SERA5*$_{synth}$ coding sequence (green), extending into the 3' non-translated sequence and terminal poly(A) tail. The TAG stop codon is indicated. The position and sequence of the *SERA5*$_{synth}$-specific primer (SERA5_3R2) used for the second step of the semi-nested RACE PCR reactions is indicated in red. The downstream *loxP* site is shown in yellow, whilst the sequences of the *Pbdt* 3' UTR (clone 3E9) and the inverted *hsp86* 3' UTR (sub-clone 1G5DiCre) are shown in pink and blue respectively. Positions of identified polyadenylation sites are indicated. Alignments were performed using Clustal W, with gaps introduced to maintain alignment where RACE products differ from the DNA sequence (probably due to proof-reading errors in the reverse transcriptase-mediated or PCR steps of the 3' RACE reactions).

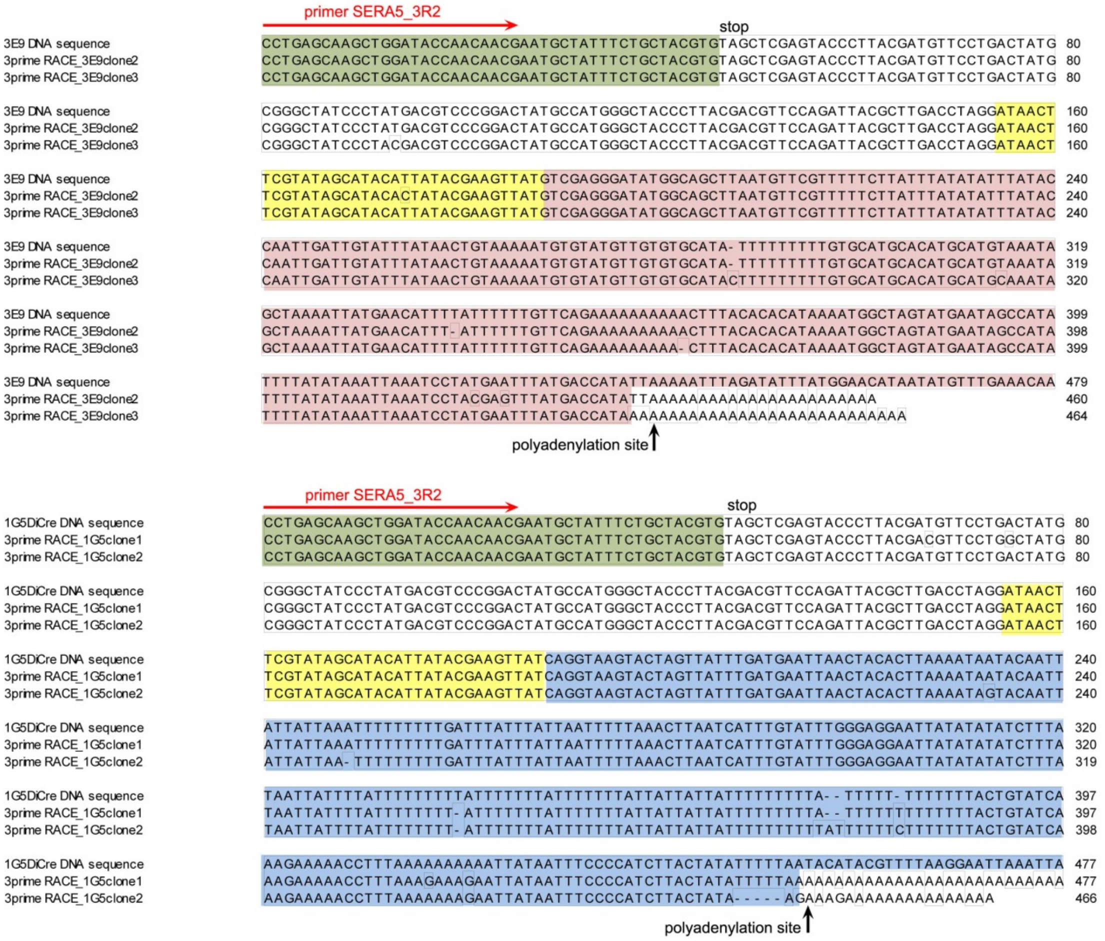

Fig. S2. Isolation of a population of WR99210-resistant parasites from rapamycin-treated 2E9 and 3E9 clones. Clones 2E9 and 3E9 were rapamycin-treated then transferred to medium containing WR99210, as described in the main manuscript . Most of the parasites rapidly died, as described in Fig. 4 of the main manuscript, but prolonged culture (>28 days) resulted in the isolation of drug-resistant parasites, called 2E9WR and 3E9WR. These were synchronised, then treated again ± rapamycin in parallel with the original 2E9 and 3E9 clones. 40 h later, genomic DNA prepared from the parasites was analysed by PCR as described in Fig. 2 (main manuscript) using primers designed to detect the intact modified SERA5 locus as well as the predicted excision event (see Fig. 1, main manuscript). Efficient, rapamycin-dependent excision was observed in the 2E9 and 3E9 clones, as expected. In contrast, the 2E9WR parasites showed some pre-existing excision, suggesting that they were the results of low-level anomalous excision that had not resulted in removal of the *hdhfr* cassette (explaining their resistance to WR99210). No excision was detected in the 3E9WR parasites, suggesting that they correspond to 'non-excisers' that could arise from spontaneous mutations in the DiCre cassette or *loxP* sites. The genomic structure of the 2E9WR and 3E9WR parasites was not further investigated.

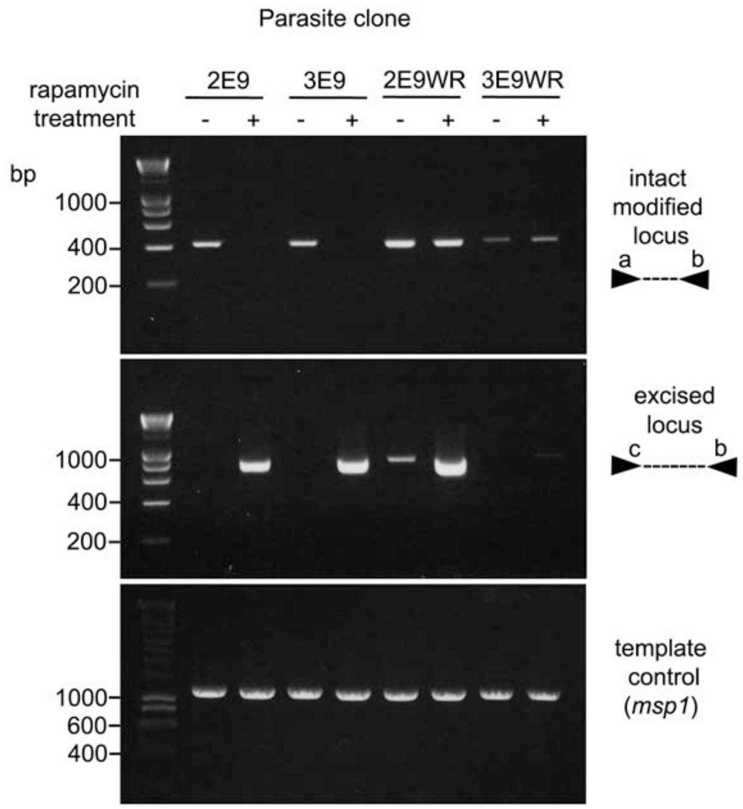

Table S1. Oligonucleotide primers used in this study

Primers	Sequence
+S5endogHpaI	GGGGTTAACGTACGGGAACAGTTAGAGGAG
-S5endogClaI	TATTATCGATAGATTCTGTTAATTTATATTCAG
+S5Seq1021	GCGATATTCCGGAAAAATGCG
-S5stopXhoI	ACTCGAGCTACACGTAGCAGAAATAGCATTC
EWmock1	ATCTGCAGGAATTCCATTTTGTAAAAAAAATTAAAATATATTTAT
EWmock2	ATAAGCTTTGATATATTTCTATTAGGTATTTATTATTATAAAATATAAATC
EWCRE59For	ATGAATTCCCTTTTTCGACAAAATGGC
EWCRE59Rev	ATCTGCAGTTAATCAGTTCAGCTTGCACC3
EWCAM3For	ATCTGCAGGATTTTTAAATATGCAGAATAAAATAAATAAGTAAATATC
EWCAM3Rev	ATGCGGCCGCCTTAAGGAACTTAATAAAAAAGAGGAG
EWCRE60For	ATAAGCTTCCTTTTTCGACAAAATGGC
EWCRE60Rev	ATGGTACCATCGATACGGTGATTAATTAATC
EWHSP863For	ATATCGATGTCGAGTTATATAATATATTTATGTACTCA
EWHSP863Rev	ATGGTACCGCGGCCGCACTAGTTATTTGATGAATTAACTACAC
sgS5seq4F	CCGCGGATCATGCGGTGAACATTGTGGGC
hsp86 5'_F1	TAAGCGGGGAATTCAGCGCTGGAAAGGGGCCATTGGATATATA
hsp86 5'_R1	GTGCATTAAGCTTACCGGTTTTATTCGAAATGTGGGAAG
bip_F1	CGCTCCCTCAGCGCTGGGGGCAAAAAGGAAAC
bip_R1	CCTAGCTTTTGCAGAATTCGCCGGCAAGAAGGAAGC
3HA_AvrII_LoxP	CCCTTACGACGTTCCAGATTACGCTTGACCTAGGATAACTTCGT ATAGCATACATTATACGAAGTTAT
AvrII_LoxP_PbDT3'_F	CCTAGGATAACTTCGTATAGCATACATTATACGAAGTTATGTCGA GGGATATGGCAGCTTAATGTTCG
NotI_PbDT3'_R	GCGGCCGCCTACCCTGAAGAAGAAAAGTCCG
MCS_HpaI	ACTAGTTACGTACTTAAGGTTAACGTACGGGAACAGTTAGAGGAG
U1_MCS	CGAAGTTATCAGGTAAGTACTAGTTACGTACTTAAGGTTAACGTACGGG
LoxP_U1_MCS_XL	ATTTTTTTTACAAAATGGTTATAACTTCGTATAGCATACATTA TACGAAGTTATCAGGTAAGTACTAGTTACGTACTTAAGGTTAACGTACG GGAACAGTTAGAGGAG
EndoS5_R1	CTTACAATATCTTCAGATAGGTATTTGTAACATACG
+27	CAATATCATTTGAATCAAACAGTGGT
-11	CTTTGCCATCCAGGCTGTTC
-25	CCATTGGACTAGAACCTTCAT
sgS5_seq5F	CCGCGCTGGAAAGCGCGGGCACCAGC
hsp86 3'_R1	GACTTTACTGAGACATG
CAM5'_R4	CATTTTGTAAAAAAAATTAAAATA
SERA5_US_F	CAAAATGAAGTCATATATTTCCTTG
SERA5_US_R	GTATCTTCTGTATCAACATC
MSP1_FOR	GGAACATCATCTACATCCAGTCCTGG
MSP1_REV	GGGTAATACAACTAAGGAATCATCTTCTTCG
SERA5_3R1	CGTGAACCTGTGCAACGTGAACTGG
SERA5_3R2	CCTGAGCAAGCTGGATACCAACAACG
3D7endoMSP1FOR1	CCATTTCTACAACAGAGATGG
3D7synMSP1_FOR2	CCGATCTGAAAGCCATCGACG
3D7endoMSP1_REV4	GCATTTTGTCTTGGCCAAGTTC
3D7synMSP1_REV3	GTAGAGATCCTGATGTGGGGATC
PbDT3'5'R1	GCTATTTACATGCATGTGCATGC
